# Clinical Bioinformatics: challenges and opportunities

**DOI:** 10.1186/1471-2105-13-S14-S1

**Published:** 2012-09-07

**Authors:** Riccardo Bellazzi, Marco Masseroli, Shawn Murphy, Amnon Shabo, Paolo Romano

**Affiliations:** 1 Dipartimento di Ingegneria Industriale e dell'Informazione, Università di Pavia, Via Ferrata 1, 27100, Pavia, Italy; 2 Dipartimento di Elettronica e Informazione - Politecnico di Milano, Milano, Italy; 3 Harvard Medical School, Boston, MA, USA; 4 IBM Research Lab in Haifa, Israel; 5 IRCCS AOU San Martino - IST Istituto Nazionale per la Ricerca sul Cancro, 16132, Genova, Italy

## Abstract

**Background:**

Network Tools and Applications in Biology (NETTAB) Workshops are a series of meetings focused on the most promising and innovative ICT tools and to their usefulness in Bioinformatics. The NETTAB 2011 workshop, held in Pavia, Italy, in October 2011 was aimed at presenting some of the most relevant methods, tools and infrastructures that are nowadays available for Clinical Bioinformatics (CBI), the research field that deals with clinical applications of bioinformatics.

**Methods:**

In this editorial, the viewpoints and opinions of three world CBI leaders, who have been invited to participate in a panel discussion of the NETTAB workshop on the next challenges and future opportunities of this field, are reported. These include the development of data warehouses and ICT infrastructures for data sharing, the definition of standards for sharing phenotypic data and the implementation of novel tools to implement efficient search computing solutions.

**Results:**

Some of the most important design features of a CBI-ICT infrastructure are presented, including data warehousing, modularity and flexibility, open-source development, semantic interoperability, integrated search and retrieval of -omics information.

**Conclusions:**

Clinical Bioinformatics goals are ambitious. Many factors, including the availability of high-throughput "-omics" technologies and equipment, the widespread availability of clinical data warehouses and the noteworthy increase in data storage and computational power of the most recent ICT systems, justify research and efforts in this domain, which promises to be a crucial leveraging factor for biomedical research.

## Background

Clinical Bioinformatics (CBI) can be defined as "the clinical application of bioinformatics-associated sciences and technologies to understand molecular mechanisms and potential therapies for human diseases" [1]. Being specifically focused on clinical context, CBI is characterized by the challenge of integrating molecular and clinical data to accelerate the translation of knowledge discovery into effective treatment and personalized medicine. CBI shares methods and goals with Translational Bioinformatics (TBI), which has been defined as the "development of storage, analytic, and interpretative methods to optimize the transformation of increasingly voluminous biomedical data - genomic data in particular - into proactive, predictive, preventive, and participatory health management" [2]. CBI and TBI can be thus considered as almost synonymous terms, being both related with the same set of scientific questions. In this paper we will refer to CBI, wanting to stress the clinical decision making aspects of bioinformatics, although we claim that the two terms are being used in current practice in an interchangeable manner.

More specifically, CBI is aimed at providing methods and tools to support two different decision-makers. On the one hand, it should assist clinicians in dealing with clinical genomics (biomarker discovery), genomic medicine (identification of genotype/phenotype correlations), pharmacogenomics and genetic epidemiology at the point of care (see [3] for a detailed discussion); on the other hand, it must support researchers in the proper reuse of clinical data for research purposes [4]. For this reason, together with bioinformatics problems, related to the management, analysis and integration of "-omics" data, CBI needs to deal with the proper definition of clinical decision-support strategies, an area deeply studied in the context of medical informatics and artificial intelligence in medicine. CBI is therefore at the confluence of different disciplines, and may foster the definition of a comprehensive framework to deal and manage all kinds of biomedical data, supporting their transformation into information and knowledge.

Even if the main aim of CBI is very ambitious, there is a variety of enabling factors that strongly support research in this direction. First of all, in the last few years new genome sequencing and other high-throughput experimental techniques have generated vast amounts of molecular data, which, when coupled with clinical data, may lead to major biomedical discoveries, if properly exploited by researchers.

Second, new diagnostic and prognostic tests based on molecular biomarkers are increasingly available to clinicians, thus consistently refining the capability of dissecting diseases and, at the same time, enlarging the decision space on the basis of the improved assessment of risk.

Third, the increasing online availability of the "bibliome", i.e., the biomedical text corpus, made through published manuscripts, abstracts, textual comments and reports, as well as direct-to-Web publications, has stimulated the development of new algorithms able to semi-automatically extract knowledge from these texts so as to make it available in computable formats. Such algorithms have been proved to be able to effectively combine the information reported in the text with that contained in biological knowledge repositories and are increasingly used for hypothesis generation, or corroboration of clinical findings. Their use in the clinics poses challenges, but may be a consistent and important tool to support decision-making.

Finally, the consistent growth of publicly available data and knowledge sources and the possibility to easily access low-cost, high-throughput molecular technologies has meant that computational technologies and bioinformatics are increasingly central in genomic medicine; cloud computing technology is being recognised as a key technology for the future of genomic research to facilitate large-scale translational research.

Network Tools and Applications in Biology (NETTAB) Workshops are a series of meetings focused on the most promising and innovative ICT tools and to their usefulness in Bioinformatics [5]. They aim at introducing participants to the most promising among evolving network standards and technologies that are being applied to the biomedical application domain. Each year, they are focused on a different technology or domain for which talks on basic technologies, tools, and platforms of interest, as well as real applications, are presented. The NETTAB 2011 workshop, held in Pavia, Italy, in October 2011 was aimed at presenting some of the most relevant methods, tools and infrastructures that are nowadays available for CBI.

In this paper, the viewpoints and opinions of three world CBI leaders, who have been invited to participate in a panel discussion of the NETTAB workshop on the next challenges and future opportunities of this field, are reported.

Looking at CBI from the technological side, these experts have identified three areas that need advancement and further research. These include the development of data warehouses and ICT infrastructures for data sharing, the definition of standards for sharing phenotypic data and the implementation of novel tools to implement efficient search computing solutions. In the following of the editorial we report such opinions and discuss their relevance to the field.

### ICT infrastructures for supporting clinical bioinformatics: important design features of the i2b2 system

i2b2 (Informatics for Integrating Biology and the Bedside) is an NIH-funded National Center for Biomedical Computing based at Partners HealthCare System that is an integrated framework for using clinical data for research [4].

The back end of i2b2 has a modular software design, called the 'Hive,' that manages everything having to do with how data is stored and accessed. The front end of i2b2 is the i2b2 Web client, a user interface that allows researchers to query and analyze the underlying data. The software is open source and can be extended by users once the core cells of the Hive are included and correctly configured.

To date, i2b2 has been deployed at over 70 sites around the world, where it is being used for cohort identification, hypothesis generation and retrospective data analysis. At many of these sites, additional functionality is being developed to suit the needs of the researchers.

Several aspects of i2b2 contribute to its rapid adoption by the clinical research community. The first is that it is open source and therefore not only is it free to try, but there is a built-in set of collaborators - other users - with whom to engage both to get help with any questions and to foster innovation. The open source, self-service nature of i2b2 allows investigators to try out ideas stepwise at their own pace and at no financial cost. The online documentation and community wiki are kept up-to-date and greatly assist in user support. Secondly, both the fact that it is open source and the modularity of the design enforce backward compatibility with existing research, so that it is added to the i2b2 platform and does not become obsolete.

But perhaps the key to the utility of i2b2 is the simplicity of its database design. A research data warehouse typically includes data from disparate sources, such as electronic health records, administrative systems, genetic and research data, and lab results, to name a few. The structure of the i2b2 database allows this data to be aggregated and optimized for rapid cross-patient searching in a way that is transparent to the user. The specific design and flexibility of the data model supports new research data being added to the database as it is amassed, while allowing users to construct complex queries against the multiple source systems.

**Figure 1:**
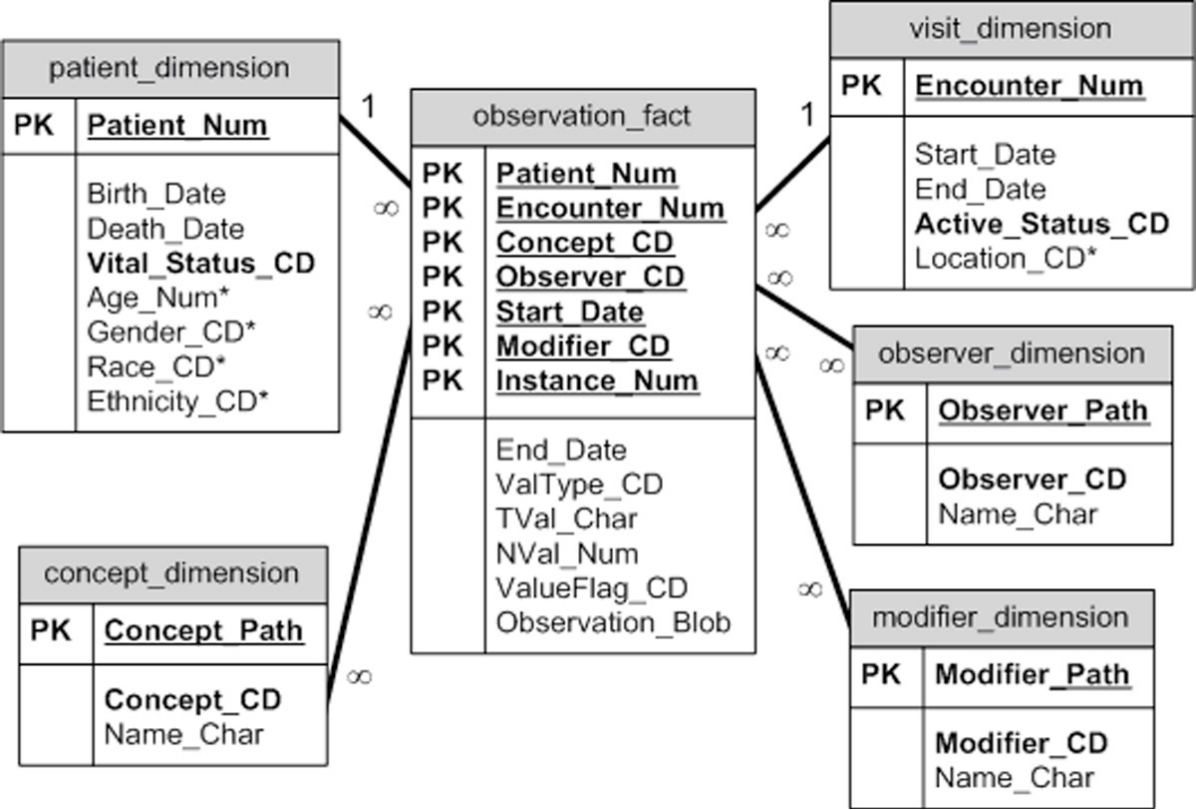
**The i2b2 star schema**.

i2b2 data is stored in a star schema, first described by Kimball [6]. A very large central fact table (observation_fact) is surrounded by and connected to the smaller dimension tables, i.e., the patient, observer, visit, concept and modifier dimensions (Figure 1). A fact is defined as an observation on a patient, made at a specific time, by a specific observer, during a specific event. Dimension tables hold descriptive information and attributes about the facts.

The star schema is optimized for analytic querying and reporting. Its design tends to mirror the way users think about and use data, which is important since users must understand what data is available in order to formulate queries. The straightforward connections between the fact and dimension tables mean that navigation through the database via joins and drilling into or rolling up dimensional data is simple and quick. The design allows the fact table to grow to billions of rows while maintaining performance. Another advantage of the fact table design is that it is well suited to handle "sparse" data; data that has many possible attributes (such as all possible medical concepts), but with only a few that are applicable. In this model, only positive facts are recorded, thus resulting in more efficient storage.

**Figure 2:**
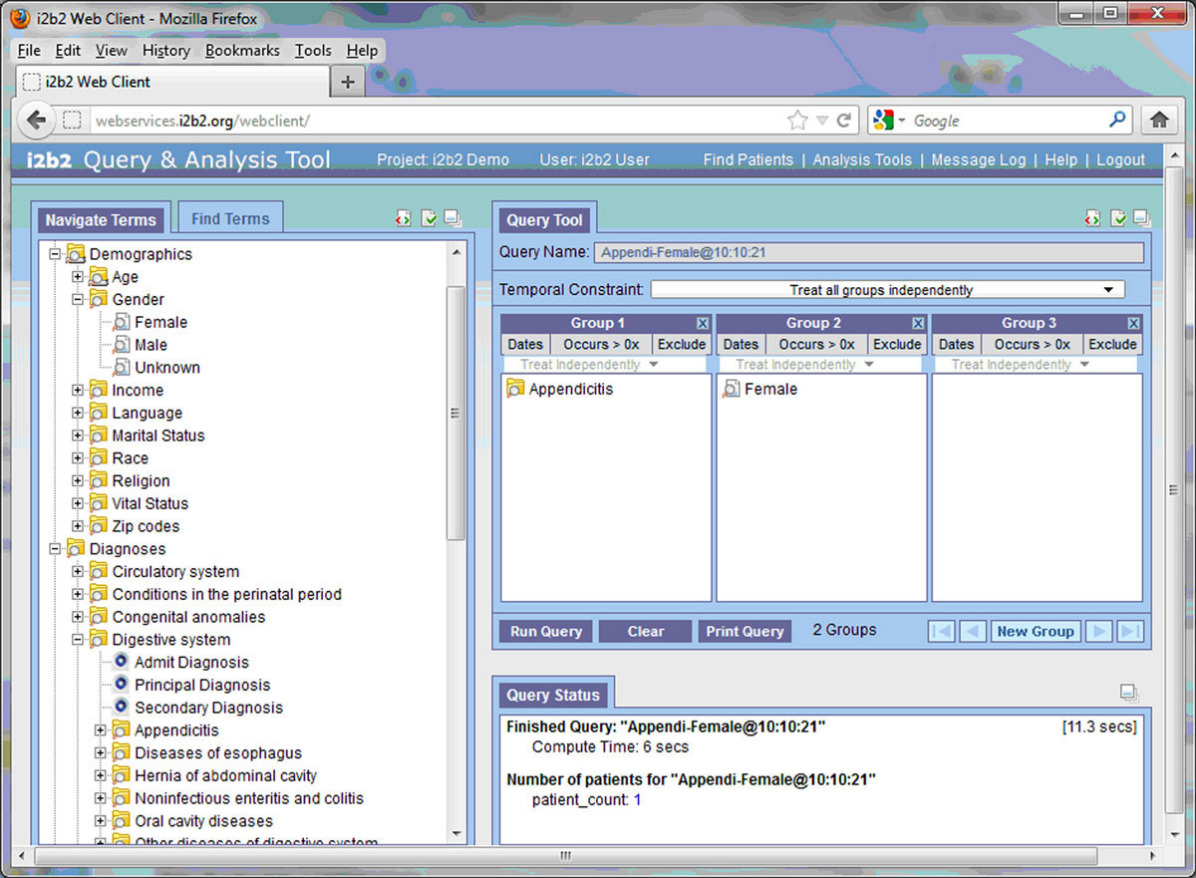
**The i2b2 Web Client is shown**. The characteristic terms (and their respective modifiers) that describe the patients in the Clinical Research Chart are shown in the tree structure on the left. The query is composed in the upper right with the logic of a "Venn-Diagram". Terms in two different Groups will be logically ANDED together, and number of patients will be shown after computation, in this case the number of patients who are both male and have had appendicitis.

Perhaps the most powerful aspect of the i2b2 database design is the design of the metadata. In i2b2, metadata is the vocabulary, all the medical terms that describe the facts in the database. Metadata is what allows users to interact with the database. A typical clinical data warehouse may have 100,000 to 500,000 concepts, including ICD-9 [7], SNOMED-CT [8], CPT [9], HCPCS [10], NDC [11] and LOINC [12] codes, as well as a host of local codes from in-house systems. Without an intuitive and easy-to-use structure, users would be stymied in understanding and using the codes. In i2b2, a hierarchical folder system is used to group the concepts. General terms are located in higher level folders, with more specific but related terms in folders and leaves underneath. The way the metadata looks in the i2b2 Web client directly reflects its structure in the table (Figure 2). A user can drill up and down in the folders in the user interface to clearly see the hierarchy and find terms of interest.

Maintaining and updating the metadata is a significant, but workable challenge. New medical codes are constantly being created, and old codes are discarded or changed. The structure of the metadata must be able to seamlessly absorb new codes while remaining backward compatible with old coding schemes. The hierarchical classification scheme of i2b2 makes it easy to map new codes to existing folders and to create new folders as needed. Entire new coding systems can be added just by creating a new folder. Discarded codes can remain in the hierarchy next to newer ones and used to reference older data, or hidden to discourage their usage in new queries.

One goal of i2b2 is to help integrate data from the many different sources that exist in modern day healthcare institutions in order to present a comprehensive view of patient care for research. The simple and intuitive design of the i2b2 database enables users to construct complex queries over these disparate data sources.

### Using the new generation of Healthcare and Life Sciences standards for Personalized Medicine

The success of Personalized Medicine (PM) at the point of care is dependent on the effective use of PM knowledge (e.g., pharmacogenomic interpretation of somatic mutations in tumor tissues) while considering the complete patient's medical history (e.g., other diseases, medications, allergies, and genetic mutations).

In order for PM knowledge to be effectively applied to the patient medical records, representations of data and knowledge need to be standardized due to the heterogeneity of their original formats. Both data and knowledge are generated nowadays by a variety of sources, each of them using proprietary formats and idiosyncratic semantics, often not represented explicitly (for example, when contextual data is unstructured and thus cannot be parsed by decision support applications).

Interpretation of clinical data typically starts at parsing the metadata, e.g., the predefined schemas of clinical information systems. However, these schemas (most often relational) cannot accommodate the complexity of contextual data representation. Thus, it is important to have a richer language allowing the explicit representation of patient-specific context of each discrete data item and of how it relates to other data items, as well as how it fits within the entire health history of an individual.

Dispersed and disparate medical records of a patient are often inconsistent and incoherent. A patient-centric, longitudinal electronic health record (EHR) based on international standards (e.g., CEN EHR 13606 [13]) could provide a coherent and explicit representation of the data's semantics. New PM evidences, generated by clinical research and validated in clinical trials and by data mining, should be represented in alignment with clinical data representations in a way that lends itself to PM realization. A constantly growing stream of raw data is available today in both research and clinical environments, e.g., DNA sequences and expression data along with rare variants and their presumed affected function, as well as sensor data along with deduced personal alerts.

The representation of such raw data should adhere, as much as possible, to common and agreed-upon reference models (e.g., HL7/ISO RIM - Reference Information Model [14] or the openEHR RM - Reference Model [13]) that provide unified representations of the common constructs needed for health information representation. For example, any observation could be represented in the same way in terms of its attributes, such as id, timing, code, value, method and status, but more importantly, using the same reference models could lead to the standard representation of clinical statements (e.g., "observation of gall bladder acute inflammation indicated having a procedure of cholecystectomy", or "EGFR variations cause resistance to Gefitinib"), where implicit semantics can become explicit and thus processable by decision support applications.

The abovementioned reference models can underlie the logical models of health data warehousing. Such warehousing could maintain the richest semantic representation of data and knowledge in a way that is also interoperable with other information systems. Performing specific tasks, such as summarizing patient data or analyzing cohort data in research studies, needs more optimized representations of the data and knowledge persisted in warehouses. Data marts are such optimized representations, and multiple data marts could be derived from a single warehouse. For example, the star schema underlying the i2b2 framework (see Figure 1) could be seen as a generic data mart for translational research that could be based on data exported from a standardized data warehouse maintained by a single health organization or across organizations, such as in the case of clinical affinity domains or integrated delivery networks.

In many cross-enterprise warehousing efforts, the main format used to convey patient data is the Clinical Document Architecture (CDA) standard [15]. CDA documents strike a balance between physicians' narratives and structured data in order to facilitate the gradual transition from unstructured clinical notes to standardized and structured data. The same transformation should also take place in knowledge representations, from scientific papers in natural language to structured knowledge, for example.

The efforts to apply Natural Language Processing (NLP) to health information could be connected to healthcare information technologies through standards like CDA that uses the clinical statement concept. The NLP fundamentals can be reduced to the clinical statement constituents and the CDA can thus be a good "catcher" of the results of NLP running over unstructured health information.

### Search and extraction of relevant information from big data amounts

The continuously increasing amount of available data poses significant technological and computational challenges, both to their management (collection, storage, integration, preservation) and effective use (access, sharing, search, extraction, analysis). This issue is becoming predominant in several fields and it is being addressed in different ways, according to each specific field peculiarities.

The Web is a paradigmatic field for this aspect. A rapidly growing mass of data is flooding the Web. Yet, leveraging on the typical linked nature of Web data, technological and computational advancements are preventing (at least for now) drowning by Web data. Automatic robots have been implemented to crawl the Web resources, collect their huge key data and store them in powerful database management systems. Effective indexing and ranking techniques, such as the Google PageRank [16], have been implemented to efficiently catalogue and sort Web resources according to their key data and likely relevance. This enables Web search engines to provide lists of items which often include among their top 10 or 20 items the one(s) that can reasonably answer numerous, yet simple, user search questions.

Such ability, which is tremendously boosting the Web as an extraordinary easy-to-use source of information, is based on the assumption that user searches are mainly aimed at finding "*at least one*" or "*the most evident*" item that can answer his/her question. Current Web search technologies are not enough when search questions either become more complex, simultaneously involve different topics, or require the retrieval of most of (if not all) available items regarding the question, possibly ordered according to different user-defined features. Furthermore, only an estimated limited part of all data accessible through the Web can actually be found by current search engines: the vast "*deep Web*", including dynamic pages returned in response to a query or accessed through a form, resources protected by password, sites limiting access by using various security technologies (e.g., CAPTCHAs), and pages that are accessible through link-produced scripts, remains unrevealed.

Especially in the CBI field, the amount of collected data is continuously and rapidly increasing, in particular with the recent collection of -omics data. Also, compared to the Web, the current ability of extracting relevant biomedical information and of answering even common CBI questions is far less, due to many reasons.

First, the biomedical-molecular data - which are of various types - are stored in several different formats within systems that are distributed, heterogeneous, and often not interoperable. Furthermore, a lot of important information is subjectively described in free texts, within chief complaints, discharge letters, clinical reports or referrals, which are intrinsically unstructured. The adoption of electronic medical or health records can significantly enhance the availability and sharing of clinical data and information, which are still only on paper in very many healthcare sites. Yet, the digitalization of health data alone is far from sufficient; having clinical reports and referrals in PDF format is evidently not enough to solve the information extraction and question answering issues. A standard data and information representation according to a shared reference model has to be adopted, together with controlled terminologies and ontologies to objectively describe medical and biomolecular findings. Moreover, the use of advanced Natural Language Processing techniques suited for the clinical domain to extract and structure information from previous medical textual descriptions can also greatly help.

Second, usual biomedical-molecular questions are generally more complex than Web search questions. They often involve more types of data, as well as topics with usually several attributes. In many cases, retrieving only a few of the items related to a biomedical-molecular search question, or even the K top items according to some user-defined ranking, may not be enough for a proper answer, which can instead require the exploration of all available items and their attributes.

Advanced search computing techniques are being developed to answer complex, multi-topic Web search questions involving the integration of possibly ranked partial search results [17]. These techniques can also be applied in the CBI domain to tackle such issues, at least partially. Yet, the complex and heterogeneous nature of the biomedical data, as well as the multifaceted structure of the clinical settings, pose formidable technological and organizational challenges for the effective management and use of biomedical-molecular data. In particular, integrated search and retrieval of bio-data, and their comprehensive analysis towards extraction of relevant information [18] and inference of biomedical knowledge, constitute some of the major challenges for the present and future of CBI, with a potential remarkable impact on the advancement of clinical research and patient treatment.

## Conclusions

CBI goals are ambitious, but many factors, from the availability of high-throughput "-omics" technologies and equipment, allowing identifying "individual" genomes and proteomes, to the incredible increase in data storage and computational power that is allowed by most recent ICT systems, justify research and efforts in this domain.

In this paper, we have reported some points of view on the current and future challenges in this domain that were discussed in a panel session at the NETTAB 2012 workshop on Clinical Bioinformatics.

First, we presented what we believe are the most important design features of a CBI-ICT infrastructure, by taking into account some achievements of the i2b2 system. Data warehousing is essential in CBI because of the great amount of clinical and biomedical information that needs to be generated and managed within health organizations. Indeed, CBI depends on information that is gathered from single individuals, usually patients, and thus it cannot exclusively depend on general population or species oriented databases that are available on-line from main data providers. On the contrary, these general resources may only be used as a general reference, while the most important data is provided by individual's clinical and molecular information.

Some of the most relevant features of a data warehouse for CBI have been identified by examining the i2b2 experience. Simplicity of the database schema is a key factor, facilitating the modularity and flexibility of the system, that support its continuous development and improvement, and making optimization of queries and searches possible. Modularity is indeed essential also because of the various and heterogeneous data and sources that may be usefully included in the data warehouse, thus leading to a multiplicity of goals and application domains for the system.

The open source approach is also extremely important, since it is able to fully exploit collaboration among users both for software development and for new features design. Collaborative development is especially important for the maintenance and update of shared metadata that, being used by the users as the main means to interact with the database, determine in fact its real usefulness and success.

Of course, individuals are moving, and information is being accumulated in many health organizations and information systems, that need to interoperate so that all possible information on each patient is made swiftly available when it is needed. This is a precondition for the clinicians in order to be able to deal at the point of care with all needed information for a proper, molecular-enabled, diagnosis, prognosis and optimized treatment selection.

Moreover, CBI data analysis may be greatly facilitated and improved when the population under analysis is the greatest possible. Such clinical-related processes as biomarker discovery and identification of genotype/phenotype correlations may only be carried out when a sufficient amount of data is available. So, interoperation of information systems should support both integration of data on single individuals and coming from many patients. Hence, it is of extreme relevance.

In this paper, we have therefore also faced the interoperability issue, and we have discussed about some of the most recent standards for data modelling and data interchange and their possible use in CBI to overcome heterogeneity of original data and knowledge formats, as well as modelling of arising information. In this case, international standards exist and should be adopted, with the provision that new evidence arising as a result of genomic medicine efforts be also properly included. We also highlighted that the application of a shared reference model could lead to a standard, semantics rich, processable representation of clinical statements.

CBI is not limited, however, to the analysis of the information on a given individual by the health care personnel that provide him/her assistance. As previously said, it must also support researchers in the reuse of clinical data for research purposes. Many new applications are being developed by researchers in the field, who can largely benefit from making access and searching information resources through Web services. It is often from such free access to data that new associations may be identified, possibly leading to hypothesis for new biomarkers validation and assessment.

In this paper, we have therefore also tried to point out which currently are the main difficulties in making access and searching CBI related information sources.

First, we addressed the idea that the adoption of common data models could be the best starting point for the implementation of a set of data marts, optimized representations of data included in warehouses for performing specific research tasks. New data marts, each devoted to a different task, could easily be created and made available.

Current techniques and technologies aimed at searching data on the Web, even the most advanced, do not seem completely adequate for CBI needs, where queries are complex, involving many data sources simultaneously and, often, requesting from each resource more results than the first that are usually returned. One of the most demanding issues remains access to a lot of information that is included, and subjectively described, in free texts, which are intrinsically unstructured. The use of controlled terminologies and of ontologies, whenever possible, together with the adoption of NLP tools suited for the clinical and biological domains can indeed support extraction of information from medical textual descriptions.

We finally moved to the issue of searching and extracting information from big data amounts. Queries which are relevant in CBI often require retrieving result sets bigger than usual and the exploration of all available items and their attributes because of possible correlations among data in the results that could change, even sensibly, their relevance to the overall query. Of particular interest are those advanced search computing techniques, which are aimed at integrating ranked search results from multiple sources.

The integrated search and retrieval of CBI data from multiple sources and its comprehensive analysis constitute in our opinion one of the biggest challenges for the future. The NETTAB 2013 workshop will be devoted to this theme.

## List of abbreviations used

CBI: Clinical Bioinformatics; CDA: Clinical Document Architecture; CEN: Comité Européen de Normalisation (European Committee for Standardization); CPT: Current Procedural Terminology; EHR: Electronic Health Record; HCPCS: Healthcare Common Procedure Coding System; HL7: Health Level 7; i2b2: Informatics for Integrating Biology and the Bedside; ICD-9: International Classification of Diseases rel. 9; ISO: International Standard Organization; LOINC: Logical Observation Identifiers Names and Codes; NDC: National Drug Code; NETTAB: Network Tools and Applications in Biology; NIH: National Institute of Health; NLP: Natural Language Processing; RIM: Reference Information Model; RM: Reference Model; SNOMED: Systematized Nomenclature of Medicine; TBI: Translational Bioinformatics.

## Competing interests

The authors declare that they have no competing interests.

## Authors' contributions

RB and PR conceived the work, wrote the background and introduction and drafted the conclusions. MM, SM, and AS contributed by identifying and specifying issues in one of the three main chapters, respectively on data search and extraction, data warehouse platform, and standardization and interoperability. All authors revised, discussed, and amended the manuscript and approved its final version.
